# Tampering by office-based methadone maintenance patients with methadone take home privileges: a pilot study

**DOI:** 10.1186/1477-7517-4-15

**Published:** 2007-10-30

**Authors:** Michael Varenbut, David Teplin, Jeff Daiter, Barak Raz, Andrew Worster, Pasha Emadi-Konjin, Nathan Frank, Alan Konyer, Iris Greenwald, Melissa Snider-Adler

**Affiliations:** 1 Ontario Addiction Treatment Centres, Canada

## Abstract

Methadone Maintenance Treatment (MMT) is among the most widely studied treatments for opiate dependence with proven benefits for patients and society. When misused, however, methadone can also be lethal. The issue of methadone diversion is a major concern for all MMT programs. A potential source for such diversion is from those MMT patients who receive daily take home methadone doses. Using a reverse phase high performance liquid chromatography method, seven of the nine patients who were randomly selected to have all of their remaining methadone take home doses (within a 24 hour period) analyzed, returned lower than expected quantities of methadone. This finding suggests the possibility that such patients may have tampered with their daily take home doses. Larger prospective observational studies are clearly needed to test the supposition of this pilot study.

## Introduction

When properly prescribed and used, methadone is an effective and safe medication in the treatment of opioid dependence and chronic pain. Prescribed methadone in adequate doses reduces cravings, prevents the onset of withdrawal, is not intoxicating or sedating, and its use does not interfere with normal activities of daily living [1,2]. In addition, methadone maintenance treatment significantly lowers illicit opioid drug use, reduces crime, and enhances social productivity [3].

The regulation of methadone varies across the world, with tighter controls in the USA, Canada and Australia [4]. In the Province of Ontario, supervised dosing is an essential component of MMT, and under certain circumstances, the prescribing physician may authorize methadone doses to be consumed by the patient without supervision, that is, by way of take home doses. Such circumstances include when patients demonstrate clinical stability, namely, the social, cognitive and emotional stability necessary to assume responsibility for the care and safeguarding of methadone, and use it only as prescribed [5]. Clinical stability also includes the elimination of sustained problematic drug or alcohol use and demonstration of mostly negative urine drug screens, a stable methadone dose, housing, employment, and/or a stable support system, and adherence to the methadone treatment agreement and program.

Potential benefits of take home doses include improved retention in treatment for existing patients, making MMT more attractive to new patients, rewarding patients for abstinence or compliance with treatment, and giving patients more control over some aspects of their treatment. In addition, the quality of life may be improved through the reduction in daily attendance at a MMT clinic [4].

However, while the privilege of take home doses has many potential benefits, it is not without potential problems. The issue of methadone diversion is a major concern for all MMT programs, as there is a substantial black market for such prescription drugs [1,6,7,3]. Given the extreme potency of methadone, it could be lethal to those who do not have the tolerance foropioids. Related to this, numerous studies have shown that the majority of methadone-related deaths have been directly related to illicit methadone diversion, and that a large percentage of those cases were not enrolled in a MMT program [5,8,2-11,6].

Fountain et al (2000) [6] point out that if prescribed methadone was consumed under strict supervision, then diversion would be minimal. However, even where prescribed methadone consumption is supervised, the sale of "spit backs" (i.e., where patients hold methadone in their mouths and spit it up or regurgitate it) can occur. Supervised doses refers to patients being supervised either by the pharmacist, physician or nurse at the clinic, the importance of which is to assure that patients take their full methadone dose, and do not divert portions of their doses to opiate-naïve individuals, with all of the associated risks.

While restrictive policies might reduce methadone diversion, they might also reduce treatment retention and increase mortality by increasing the population of untreated opioid users [5]. Therefore, methadone prescribers will need to find a balance between strengthening the self-responsibility for as many MMT patients as possible, while at   the same time, making MMT as secure as possible for both those in such treatment programs as well as the general population [10].

## Methods

### Objectives

This pilot study was undertaken to determine the need for a larger observational study to measure the extent to which patients with take-home methadone privileges may possibly tamper with their take home methadone doses and, therefore may be a potential source for methadone diversion.

### Ethics

A Quality Assurance Method (QAM) was utilized for this study in order to determine if program expectations were being met, thereby providing a baseline for improvement. As a purely observational study, this did not alter patient care in any way. As such, because identifying patient data was not revealed, this study was exempt from formal ethics review by our local Ethics Review Board.

### Population and setting

All nine patients were actively enrolled in an outpatient MMT program. All met the DSM-IV-TR diagnosis for Opioid Dependence. Six of the nine patients were male, with an average age of 34 years old. Six patients were married; the remaining three were single, common-law, and divorced. The average methadone dose was 129 mg/100 ml. All nine patients had achieved either Level 5 or Level 6 status in the Clinic. This means that such patients have been on the MMT program for a minimum of six months and during which time they underwent supervised twice weekly urine testing that produced substance-free samples. All urine samples were analyzed using the NOVX iMDx Analyzer (quantitative or qualitative analysis with industry standard or customized cutoffs). In addition, in order to achieve such Level 5 or Level 6 status, MMT patients had to have been deemed by their methadone doctors as "clinically stable", as defined by The College of Physicians & Surgeons of Ontario Methadone Treatment Guidelines, 2005 [5]. Such a definition includes the elimination of sustained problematic drug or alcohol use and demonstration of mostly negative urine drug screens, a stable methadone dose, housing, employment, a stable support system, and adherence to the methadone treatment agreement and program. Out of those nine patients, three received five daily take-home methadone doses per week (Level 5 status), and six received six daily take-home methadone doses per week (Level 6 status).

### Standard care

In keeping with standard clinic practice, patients with take home doses are randomly called and asked to return to the clinic within 24 hours with all of their remaining methadone take home doses in order to confirm compliance with prescribed dosing and to rule out the possibility of the potential for diversion. In an attempt to reduce the likelihood of other possible alternatives, the doses dispensed to the pilot study patients were dispensed from the same pharmacy, with a "triple check" system in place in order to try and minimize and/or avoid any discrepancy in prescribed and/or dispensed doses. This dispensing method is used across all doses dispensed by the community pharmacy.

Related to the dispensing method, all bottles are sealed in the same manner, and any spillage is avoided, or recorded if it occurs. Spilled doses would be replaced prior to dispensing to the patients. It is possible however, that spillage can occur after point of dispensing, once the doses are in the patient hands.

As inaccurate methadone dosing by pharmacies can possibly occur, additional factors were controlled for in an attempt to minimize this possibility when the quantity of methadone being measured in the returned carries was inaccurate.  These included the medium (juice) in which the methadone was dispensed, temperature at the time of dispensing, as well as the type of bottles and seals used.

The information yielded from these returns is then provided to the methadone-prescribing physician in order to determine if any management changes are necessary.

### Selection

Physicians at the clinic who were blinded to the study objectives were asked to submit the names of their patients who were scheduled for a random call back to the clinic within the following week.

### Measurement

The returned methadone doses were analyzed using a Reverse Phase High Performance Liquid Chromatography method (solid phase extraction and RP-HPLC with coupled UV detection), developed to measure total content of methadone and its enantioners in syrup samples [12]. The biochemist performing the analysis was blind to the study objectives and the identity and clinical details of each patient enrolled.

### Outcomes

The primary outcome for this study was evidence of possible tampering, as indicated by a difference between the total volumes and/or amount of methadone dispensed (expected to be present) and the amounts measured to be remaining in the carry doses within 24 hours of the call back. The secondary outcome was the number of patients with methadone missing.

## Results

Of the nine MMT patients that were randomly chosen, we found that seven of those may have possibly tampered with their take-home methadone doses. More specifically, seven of the nine MMT patients returned lower than expected quantities of methadone, while one patient returned more than the expected quantity (Table 1).

**Table 1:** Differences between volume dispensed and expected take home methadone doses

**Pt**.	**Take-Home Doses**	**Difference between volume (ml) dispensed and expected**	**Difference between amount (mg) dispensed and expected**	**Methadone Missing**
1	6	0 ml	-40.2 mg	Yes
2	6	-18 ml	-33.4 mg	Yes
3	6	0 ml	-211.2 mg	Yes
4	6	-28 ml	-137.2 mg	Yes
5	6	0 ml	0 mg	No
6	5	0 ml	-56.76 mg	Yes
7	5	0 ml	-60.0 mg	Yes
8	5	0 ml	43.32 mg (extra)	No
9	5	0 ml	-19.1 mg	Yes

## Discussion

This pilot study suggests that over three quarters of MMT patients may possibly have tampered with their daily take-home methadone doses. When followed up by their own methadone prescribing physicians as to why such discrepancies may have occurred, patient explanations included using larger amounts of methadone than prescribed and then having to purchase methadone from the street to make up for the "short-fall", or splitting their take home doses into multiple daily amounts (depending on symptoms and needs) and then trying to adjust the take home methadone doses to the original concentration (when randomly asked to bring in the take home doses). Of course, not having the same ability to measure and dilute with proper solution, results in vastly different quantities of methadone in patients' take home methadone doses.

The findings of this pilot study are somewhat bothersome in that the vast majority of the nine MMT patients who were deemed to be "clinically stable" (as defined by The College of Physicians & Surgeons of Ontario) may have potentially tampered with their daily take home methadone doses. This raises the question as to what extent other "clinically stable" MMT patients may also possibly be tampering with their daily take home methadone doses.

Methadone diversion is a dangerous practice for both patients who are self-adjusting and medicating their methadone regimen, and those who are on the receiving end of diverted methadone. Patients who are diverting a portion or their entire methadone dose, and are then required to consume a witnessed regular full strength dose, are at high risk of overdose. Those obtaining a diverted methadone dose and ingesting an unknown amount of methadone are also at risk of overdose.

Clearly, one of the major limitations of this pilot study was that the sample size was small, thus limiting the ability to generalize such findings to a broader office-based MMTP population. Another limitation was that this study was conducted in Canada (and specifically the Province of Ontario) and therefore such findings may highlight the differences in how other countries or jurisdictions practice methadone maintenance treatment, including the privilege of earning take home doses (carries).  In addition, this study lacked a control group.  As such, a much larger prospective observational study is strongly recommended in order to test such suppositions.

While undoubtedly there are several compelling arguments for daily take home methadone privileges, these also pose some significant health and societal risks, and the potential for increased prescription methadone diversion. Thus, it is imperative that MMT program providers maintain a balance between implementing better control measures in order to minimize methadone diversion, while at the same time, continuing to provide opiate dependent patients ease of access to MMT treatment. Such control measures can be in the form of limiting carry doses, increasing the frequency of urine testing for drugs of abuse (given the narrow window of detection time of between 1–3 days, depending on the type and class of substance), testing for Methadone Metabolites (EDDP), establishing routine "call backs" for those with large numbers of take home doses and analysis of dispensed take home doses.
